# Comprehensive epidermal growth factor receptor gene analysis from cytological specimens of non-small-cell lung cancers

**DOI:** 10.1038/sj.bjc.6604142

**Published:** 2007-12-18

**Authors:** S Savic, C Tapia, B Grilli, A Rufle, M P Bihl, A de Vito Barascud, M Herzog, L Terracciano, F Baty, L Bubendorf

**Affiliations:** 1 Institute for Pathology, University Hospital Basel, Basel, Switzerland; 2 Department of Pneumology, University Hospital Basel, Basel, Switzerland

**Keywords:** non-small-cell lung cancer, EGFR, mutation, FISH, cytology

## Abstract

*Epidermal growth factor receptor (EGFR) gene* mutations and increased copy numbers are considered as predictors of response to EGFR tyrosine kinase inhibitors (EGFR-TKI) in non-small-cell lung cancer (NSCLC). Lung cancer diagnosis is often based on cytology alone. However, almost all published data on *EGFR gene* analyses were obtained from biopsies. This study tested the feasibility of *EGFR gene* analyses on cytological specimens. Eighty-four cytological specimens from NSCLCs were prospectively analysed for *EGFR gene* mutation in exons 18–21 and *EGFR gene* copy numbers were evaluated by fluorescence *in situ* hybridisation (FISH). A FISH-positive result was defined according to the criteria by Cappuzzo *et al* established for biopsies of NSCLCs. Fluorescence *in situ* hybridisation results of cytological specimens were compared to the FISH results on matching biopsies (*n*=33). Initial diagnosis of NSCLC was solely based on cytology in 37 out of 84 (44.0%) patients. Out of 80 NSCLCs, 6 (7.5%) showed *EGFR gene* mutations. Out of 67 cancers, 45 (67.2%) were FISH positive on cytological specimens. Comparison of FISH showed a FISH-positive result in 21 out of 33 (63.6%) cytological specimens but in only 8 out of 33 (24.2%) matched biopsies. *Epidermal growth factor receptor gene* analyses are well applicable to cytological specimens. The high FISH-positive rate of NSCLC on cytological specimens contrasts with the low rate on biopsies when previously suggested criteria are used. New criteria for a positive *EGFR* FISH status to predict response to therapy with EGFR-TKI need to be defined for cytological specimens.

Despite strong therapeutic efforts, the overall 5-year survival rate of patients with lung cancer is only 15% without having improved over the last 30 years emphasising the need for new therapies (Jemal *et al*, 2006).

The epidermal growth factor receptor (EGFR) is known to play a role in the development and progression of cancer, and small molecular EGFR tyrosine kinase inhibitors (EGFR-TKI), including gefitinib (Iressa®; Astra Zeneca, Macclesfield, UK) and erlotinib (Tarceva®; OSI Pharmaceuticals Inc., Melville, NY, USA), are available (Arteaga, 2002; Meert *et al*, 2002; Schlessinger, 2002; Herbst and Bunn, 2003).

Somatic mutations within the tyrosine kinase domain of the *EGFR gene* prevail in a subset of non-small-cell lung cancers (NSCLC). These mutations are preferentially found in women, east Asians, never smokers and adenocarcinomas, often with a bronchioloalveolar histology (Fukuoka *et al*, 2003; Kris *et al*, 2003; Lynch *et al*, 2004; Miller *et al*, 2004; Paez *et al*, 2004; Perez-Soler *et al*, 2004; Eberhard *et al*, 2005; Shepherd *et al*, 2005; Tsao *et al*, 2005). Initial retrospective studies showed an average response rate to EGFR-TKI of 75% for NSCLC with *EGFR* mutations that contrasted with a low response rate of <10% for tumours with wild-type *EGFR gene* (Riely *et al*, 2006). These results were subsequently confirmed in prospective studies (Riely *et al*, 2006). Since response to EGFR-TKI is not fully restricted to patients with *EGFR*-mutated NSCLC suggests that additional molecular mechanism may be involved (Tsao *et al*, 2005). In fact, a high *EGFR gene* copy number detected by fluorescence *in situ* hybridisation (FISH) was also shown to predict improved survival after EGFR-TKI therapy (Cappuzzo *et al*, 2005; Hirsch *et al*, 2005).

A considerable fraction of NSCLCs is diagnosed solely by cytology, and the relative paucity of tumour cells on these specimens is a challenge for molecular analyses (Sequist *et al*, 2007). Since almost all of the published data on *EGFR* mutation and gene copy number analyses were made on biopsy material, the goal of this study was to test whether such *EGFR gene* analyses are feasible on cytological specimens of NSCLCs in a diagnostic setting.

## MATERIALS AND METHODS

### Cytology and biopsy specimens

A consecutive series of 84 cytological specimens with NSCLC diagnosed during November 2004 to January 2006 was entered into the study. Sixty-five specimens were from primary tumours and 19 from regional lymph node metastases of the mediastinum. The specimens included 35 transbronchial fine needle aspirates, 15 bronchial washings, 13 bronchial brushes, 5 bronchoalveolar lavages and 16 pleural effusions.

The specimens were processed according to routine procedures, using Delaunay's solution as a fixative. They were stained according to Papanicolaou and permanently mounted with coverslips.

In 33 patients, a matched biopsy of the NSCLC was available for comparative analysis. Biopsies were fixed in 4% buffered formalin, and paraffin-embedded biopsies were cut into 4 μm sections and stained with haematoxylin and eosin. In 26 of these 33 paired specimens, both cytology and biopsy were from the primary tumour sites (nine bronchial washings with nine bronchial biopsies; six bronchial brushes with four bronchial biopsies, one pneumonectomy and one pleural biopsy; five transbronchial fine needle aspirates of the lung with three bronchial biopsies and two lobectomies; four pleural effusions with two pleural biopsies, one bronchial biopsy and one lung examined at autopsy; two bronchoalveolar lavages with one bronchial biopsy and one pneumonectomy).

### Sequence analysis of the EGFR gene

Cancer cells from Papanicolaou-stained cytological specimens and from haematoxylin–eosin-stained tissue sections were selectively dissected under visual control using laser microdissection in combination with a laser pressure catapulting system according to the manufacturer's guidelines (PALM® MicroBeam, Microlaser Technologies GmbH, Bernried, Germany). Laser energy catapulted cells were collected in the cap of a 0.5 ml plastic tube containing 80 μl of 1 × PCR buffer (Applied Biosystems, Foster City, CA, USA). A 20 μl portion of proteinase K was added and incubated overnight at 56°C. The enzyme was inactivated by heating at 95°C for 10 min. In order to avoid recently described false-positive point mutations by polymerase (Marchetti *et al*, 2006), we performed two independent multiplex PCRs using 5 μl of this solution, each as a template followed by a second multiplex PCR.

The first multiplex PCR contained all forward and reverse primers for the EGFR exons 18, 19, 20 and 21 (Table 1). For the second multiplex PCR with nested and seminested primers (Table 1), 1 μl from the first multiplex PCR was used as a template.

For the first and the second PCRs, 50 PCR cycles were performed using the hot start AmpliTaq Gold polymerase (Applied Biosystems) under the following conditions: denaturation: 20 s at 95°C, annealing: 10 s at 59°C, elongation: 40 s at 72°C.

After exon amplification, unused primers were cleaned up with ExoSAP-IT (USB Corporation, Cleveland, OH, USA). After inactivating the enzyme at 80°C for 15 min, we used 0.5 μl as template for the sequencing PCR. Sequencing was performed in forward and reverse direction for every exon.

The sequence amplicons were detected by capillary electrophoresis with laser-induced fluorescence detection (3130 Genetic Analyzer, Applied Biosystems/Hitachi Inc., Foster City, CA, USA). The resulting four visualised sequences (chromatograms) per exon were analysed using SeqScape 2.5 Software (Applied Biosystems). The sequence analysis of the EGFR gene was considered evaluable if at least two chromatograms of every exon and at least one in each separate DNA isolate were readable.

### Specimen pretreatment and FISH assay

A hybridisation target area of 18 × 18 mm to 24 × 50 mm depending on cellularity was selected and permanently marked with a diamond pen. The exact locations of the carcinoma cells were saved by a relocation software (Mark&Find Module; Carl Zeiss Vision GmbH, Hallbergmoos, Germany) connected to an automated stage (Type 00-24-473-0000; Carl Zeiss AG, Oberkochen, Germany) on a Zeiss Axioplan 2 epifluorescence microscope (Carl Zeiss GmbH Jena, Jena, Germany). Before uncovering and hybridisation, a representative cell group was photographed with a digital camera (AxioCam Color, Type 412–312).

The commercially available LSI EGFR SpectrumOrange/CEP 7 SpectrumGreen dual-colour probe set (Vysis Inc., Abbott Laboratories, Downers Grove, IL, USA) was used. It includes directly labelled DNA FISH probes for the *EGFR gene* (7p12, SpectrumOrange) and the centromere of chromosome 7 (at 7p11.1–q11, SpectrumGreen). Fluorescence *in situ* hybridisation on cytological specimens was performed according to the recommendations of the manufacturer with minor modifications, as described previously (Savic *et al*, 2006).

For FISH on biopsies, we used haematoxylin–eosin-stained tissue sections using our previously described standard FISH protocol (Stadlmann *et al*, 2006). No separate procedure for destaining was required.

### Enumeration of FISH signals

Enumeration of FISH signals was performed at a magnification of × 630 after automatic relocation of the cancer cells by a relocation software in the DAPI single-bandpass filter set on a fluorescence microscope. Nuclear signal enumeration was only performed on cells with clearly defined nuclear borders and clearly visible signals. A maximum of 100 cancer cells were scored.

NSCLCs were defined as FISH positive according to previously defined criteria by Cappuzzo *et al* (2005): a FISH-positive result was defined as presence of ‘high polysomy’ (⩾4 *EGFR gene* copies per nucleus in ⩾40% of the analysed cancer cells) or of amplification (presence of tight *EGFR gene* clusters and a ratio of *EGFR gene* to chromosome of ⩾2, or ⩾15 *EGFR gene* copies per nucleus in ⩾10% of the analysed cancer cells).

### Data analysis

Statistical tests included two-tailed Fisher's exact, Pearson's *χ*^2^ and Wilcoxon signed-rank test. Statistical significance was defined as *P*<0.05. Statistical analysis was performed with JMP 5.1 software (SAS Institute, Cary, NC, USA). Receiver operating characteristic (ROC) curve analysis was performed using the statistical software R (v. 2.4.0) (Team, 2004).

## RESULTS

### Patient and tumour characteristics

Clinicopathological data of the 84 patients are summarised in Table 2. Sixty-three (75.0%) patients had an inoperable UICC (International Union Against Cancer) tumour stage IIIB, IV or a recurrence. There was no significant difference in tumour histology and tumour stage between male and female patients.

The diagnosis of lung cancer was solely based on cytology in 37 out of 84 (44.0%), and a simultaneous diagnosis of lung cancer by cytology and biopsy was made in 34 out of 84 (40.5%) patients. The remaining 13 (15.5%) NSCLC were initially diagnosed by cytology and subsequently by biopsy obtained within a median of 20 days (ranging 8–54 days) after cytology.

### EGFR gene sequencing and mutation status

Results of *EGFR* DNA mutation analysis and their associations with clinicopathological data of the 84 patients are summarised in Table 3. DNA sequencing was successful in 78 out of 84 (92.9%) NSCLCs analysed from cytological specimens. We found four *EGFR gene* mutations (one on a fine needle aspirate and three on bronchial washings). Matched biopsies were available in three of the six NSCLCs that were not evaluable for *EGFR* mutation. Sequence analysis was repeated on these tissue specimens revealing mutations in two of these, including a point mutation in exon 21 (R832H) and a silent mutation in exon 20 (F795F), respectively. The *EGFR gene* sequence of the third NSCLC was not evaluable from the biopsy specimen either. Comparison of the 78 cases evaluable for *EGFR gene* sequencing with the 6 non-evaluable ones revealed no significant differences in the mean microdissected tumour cell areas or the estimated cell counts (data not shown).

In summary, 6 out of 80 (7.5%) NSCLCs showed *EGFR* mutations: 4 diagnosed on cytological specimens and 2 on biopsy specimens. These mutations included the well-described deletion E746-A750 in exon 19 and the point mutation L858R in exon 21 as well as the previously described point mutation R832H in exon 21 and the insertion ASV 770–772 in exon 20 (Shigematsu *et al*, 2005; Giaccone *et al*, 2006).

Four of the six NSCLCs with *EGFR* mutation were from female patients.

Table 4 shows a summary of NSCLCs *EGFR* mutations, including mutation types, *EGFR* FISH results and patient characteristics.

### Estimate of the minimal number of cells needed for EGFR gene sequencing

In order to evaluate the minimal number of cells for successful *EGFR* DNA sequence analysis, we compared the quality of DNA chromatograms from exons 18, 19, 20 and 21 of the *EGFR gene* obtained from 30, 50 and 100 cancer cells. The cells were dissected from five cytological specimens from NSCLC with known *EGFR* mutation status. Four of these NSCLCs had no *EGFR* mutation and one had a point mutation in exon 21 (L585R). The best result was obtained with 100 analysed cells with 19 out of 20 evaluable chromatograms, followed by 50 cells with 18 out of 20 evaluable chromatograms. With 30 analysed cancer cells, only 12 chromatograms were evaluable. The differences in the evaluability between the different cell counts were not statistically significant. The point mutation in exon 21 (L858R) was identified with 30, 50 and 100 cells.

### EGFR gene copy number

In 75 out of 84 (89.3%) specimens, a sufficient number of cancer cells remained for FISH analysis after tumour cell dissection for EGFR gene mutation analysis. Fluorescence *in situ* hybridisation analysis was successful in 67 out of 75 (89.3%) specimens. The mean number of scored cells was 70±32.7 (ranging 12–100; 95% confidence interval (CI): 62.0–78.0). Fluorescence *in situ* hybridisation was positive according to the criteria defined by Cappuzzo *et al* (2005) in 45 out of 67 (67.1%) of the cytological specimens, including 35 out of 67 (52.2%) NSCLCs with ‘high polysomy’ and 10 out of 67 (14.9%) with amplification.

**Figure 1:**
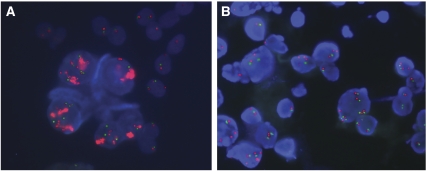
Representative images of FISH-positive results on cytological specimens (*EGFR gene*: red signals; chromosome 7: green signals). (**A**) Amplification of the *EGFR gene* with tight *EGFR gene* clusters; (**B**) high polysomy of the *EGFR gene* in the cancer cells. FISH=fluorescence *in situ* hybridisation; EGFR=epidermal growth factor receptor (see online version for colour figure).

Representative images of FISH-positive results are illustrated in Figure 1.

A matched biopsy specimen was available in 33 patients for comparative FISH analysis. The mean number of scored cells was significantly higher in the biopsy specimens compared to the cytology specimens (mean 88.6±17.6: ranging 43–100; 95% CI: 82.4–94.9 *vs* mean 66.1±31.6: ranging 12–100; 95% CI: 54.9–77.3; *P*<0.01). Fluorescence *in situ* hybridisation was positive in 21 (63.6%) of these 33 cytological specimens, including 19 (57.6%) cases with ‘high polysomy’ and 2 (6%) cases with amplification. Fluorescence *in situ* hybridisation on biopsy specimens was positive in only 8 out of 33 (24.2%) NSCLCs, including 5 (15.1%) with ‘high polysomy’ and 3 (9.1%) with amplification (Table 5). The substantial difference in the FISH results between the matched cytologies and biopsies was almost unchanged when the analysis was restricted to the primary tumours (*n*=26) after exclusion of the regional lymph node metastases (*P*=0.01). Thus, the difference cannot be explained by a change of FISH status during progression to metastasis. The number of scored cells remained significantly higher in the biopsy specimens compared to the cytology specimens.

Two amplified NSCLCs showed amplification both in the cytology and in biopsy specimen. In another NSCLC *EGFR gene* amplification was found in the biopsy but not in the cytological specimen. A review of the slides revealed that the tumour cells in the biopsy were much less differentiated than the cells in the cytological specimen. This suggests that the discrepancy between cytology and histology in this patient could be due to tumour heterogeneity.

In order to synchronise the FISH status of cytological and histological specimens, we tuned the threshold of a positive FISH result for cytological specimens using ROC curve based on the 33 cases with matched specimens (Linden, 2006). Changing the number of gene copies and the minimal percentage of cells in cytological specimens would identify the ideal threshold with maximal sensitivity and specificity. Applying the set of parameters used for definition of ‘high polysomy’ on biopsies (⩾4 copies in ⩾40% of cells) to cytology resulted in a sensitivity, specificity, positive predictive value (PPV), negative predictive value (NPV) and *κ* of 1.0, 0.45, 0.33, 1.0 and 0.26, respectively. The optimised parameters obtained by the ROC curve for ‘high polysomy’ on cytological specimens were ⩾5 copies in ⩾70% of cells. The sensitivity, specificity, PPV, NPV and *κ* were 0.67, 1.0, 1.0, 0.92 and 0.76 respectively.

Comparison between mutation and FISH status showed that four of the six *EGFR gene*-mutated NSCLCs had a FISH-positive result, including two with ‘high polysomy’ and two with amplification. *Epidermal growth factor receptor gene* analysis was mostly performed on cells from Papanicolaou-stained specimens but also worked well in cases in which only immunostained slides were available. The *EGFR gene* sequence was evaluable in 5 out of 6 specimens and FISH in 7 out of 10 specimens after immunocytochemistry for TTF-1, or BerEp4, or cytokeratine 7, or cytokeratine 20.

## DISCUSSION

In this study, we demonstrate that a comprehensive *EGFR gene* analysis for prediction of response to EGFR-TKI in NSCLC is well feasible in routinely processed cytological specimens. In most patients, small biopsies or cytological specimens must suffice for both morphological diagnosis and predictive marker analyses. Importantly, as in our series, NSCLC is often diagnosed by cytology alone (Rivera *et al*, 2003). In contrast, most previous studies on *EGFR gene* mutations or copy number analyses in lung cancer were based on resection specimens or biopsy material. The clinical effect of EGFR-TKI in a proportion of patients with NSCLC is associated with several molecular alterations, including *EGFR* mutations and increased copy number of the *EGFR gene*, although the relative importance of the alterations remains controversial (Cappuzzo *et al*, 2006; Dziadziuszko *et al*, 2006; Riely *et al*, 2006).

We found *EGFR* mutations in 7.5% of the patients. This is in accordance with previous studies in Caucasian patients where the prevalence ranged from 4.5 to 14.1% (Kosaka *et al*, 2004; Marchetti *et al*, 2005; Sasaki *et al*, 2005; Tokumo *et al*, 2005; Yang *et al*, 2005).

For mutation analysis, enrichment of morphologically identified cancer cells is crucial in order to avoid dilution of tumour DNA with non-mutated normal DNA from benign cells that often outnumber the malignant cells. This can readily be achieved by laser microdissection. We were able to determine the DNA sequence from as few as 30 tumour cells.

Cappuzzo *et al* (2005) were the first to propose a strong predictive role of *EGFR gene* copy number for response to therapy with EGFR-TKI in NSCLC. They found a positive *EGFR* FISH status defined as ‘high polysomy’ or ‘amplification’ in 32.4% of biopsies from 102 patients with NSCLC being an independent predictor of response to erlotinib. The importance of *EGFR gene* copy number for therapy response to EGFR-TKI has subsequently been confirmed by several studies (Bell *et al*, 2005; Eberhard *et al*, 2005; Tsao *et al*, 2005; Hirsch *et al*, 2006). However, all of these previous studies examining *EGFR* FISH were based solely on histological tissue sections and did not consider cytological specimens. This may have been due to difficulties in differentiating reactive cells from admixed cancer cells after hybridisation. In fact, we regard automated relocation of carcinoma cells identified in Papanicolaou-stained specimens as a prerequisite of FISH analysis in cases in which cancer cells are admixed with a high proportion of benign cells. Disregarding cytological specimens in a previous randomised study on erlotinib in 731 NSCLCs may be one of the reasons why the percentage of specimens used for mutation and FISH analyses was as low as 27 and 30%, respectively (Shepherd *et al*, 2005; Tsao *et al*, 2005).

Applying the criteria of Cappuzzo *et al* in our study revealed a high FISH-positive rate of NSCLC in cytological specimens (63.6%) that contrasted with a low rate in matched biopsy specimens (24.2%). The latter was in line with the prevalence of FISH positivity found in previous studies (Table 6). The discrepancy between cytology and histology is explained by the fact that a proportion of the cell nuclei on biopsy tissue sections are truncated, leading to a lower number of gene signals per nucleus. In contrast, the cell nuclei on the cytological specimens remain intact and contain the true number of gene copies. The discrepancy remained almost unchanged when analysing only primary tumours, dispelling gene copy differences due to tumour heterogeneity between primary tumours and locoregional metastases. Thus, thresholds of FISH positivity defined on histological sections cannot directly be translated to cytological specimens. This is especially true for low-copy-number gains such as the category of ‘high polysomy’, where at least four signals per nucleus are required in at least 40% of the cells. A proportion of tumour cells with three signals or two signals in histological sections would in fact contain four signals if the nuclei were not truncated. Thus, some of these cases would cross the threshold for FISH positivity if the nuclei were intact. In contrast, nuclear truncation does not affect the ability to detect high-level amplifications, as previously shown in cytohistological comparisons of *HER-2* FISH analysis in breast cancer (Bozzetti *et al*, 2002; Gu *et al*, 2005). This concurs with our results where the difference between histology and cytology was greatest in the category of ‘high polysomy (57.6 *vs* 15.2%, *P*<0.01), whereas the prevalence of amplification was comparably low (6 *vs* 9.1%). An optimised threshold of ⩾5 gene copies in ⩾70% of cells as calculated from ROC curve analysis could substantially improve the specificity and PPV of FISH in cytology when the FISH result of histology was used as a gold standard. However, this was at the cost of reduced sensitivity to a low 67%. There have been previous attempts to compensate for the discrepancies between whole-cell preparations and tissue sections for more accurate enumeration of FISH signals (Berezuk *et al*, 2001). However, the proposed correction factors based on the cell diameter are limited by the variability of nuclear size and shape in cancer cells, which makes this approach insufficient in a diagnostic setting.

Cytological specimens and matched biopsies are not always from the identical tumour area. Therefore, it is possible that the difference in *EGFR* FISH status between cytology and biopsy in this study might be partly attributable to heterogeneity. However, it is very unlikely that tumour heterogeneity had a unidirectional effect towards a 3.8-fold higher rate of ‘high polysomy’ in cytology than in biopsy (57.6 *vs* 15.1%). It is similarly unlikely that the lower mean number of tumour cells available for *EGFR* FISH analysis in cytological specimens selected for ‘high polysomy’. A study on a larger series of matched histological and cytological specimens is under way to investigate the impact of tumour heterogeneity and cell count on the *EGFR* FISH status, and to define better criteria of a positive *EGFR* FISH result in cytological specimens.

It is becoming increasingly important to analyse prognostic and predictive markers in tumour biopsies as exemplified by *EGFR gene* analysis in *NSCLC*. This is also true for cytological specimens that are often the only source of cells from primary tumours or distant metastatic sites. When dealing with limited cell material in a diagnostic setting, the morphological diagnosis still has priority over molecular analyses. Here, we show that comprehensive molecular data can be obtained even from few cancer cells on diagnostic cytological specimens, which is facilitated by laser microdissection and automated relocation. Our data challenge the recently expressed opinion that the ‘widespread use of mutational analysis is currently hindered by the routine use of very small fragments of tissue to establish the diagnosis of NSCLC’ (Riely *et al*, 2006). Importantly, standard staining such as Papanicolaou does not interfere with FISH and DNA analysis by PCR, and these methods can even be applied to previously immunostained specimens. This is important since immunocytochemical assessment of proteins such as EGFR or p-Akt may also be of value in predicting response to EGFR-TKI (Cappuzzo *et al*, 2004; Tsao *et al*, 2005). Notably, these techniques and tools are not restricted to *EGFR* analysis and also set the stage for the analysis of future therapeutic targets.

## Figures and Tables

**Table 1:** Primers used for the *EGFR gene* mutation analysis for the first and the second PCR

	**Primers**	
**EGFR gene**	**Forward**	**Reverse**
*Exon 18*		
First PCR	GCATGGTGAGGGCTGAGGTGA	CCCCACCAGACCATGAGAGGC
Second PCR	ACCCTTGTCTCTGTGTTCTTGTCCC	GCCCAGCCCAGAGGCCTGTG
		
*Exon 19*		
First PCR	TGCCAGTTAACGTCTTCCTTC	CCACACAGCAAAGCAGAAAC
Second PCR	AACGTCTTCCTTCTCTCTCTG	CCACACAGCAAAGCAGAAAC
		
*Exon 20*		
First PCR	CCACCATGCGAAGCCACACTGA	TCCTTATCTCCCCTCCCCGTATCTC
Second PCR	CCATGCGAAGCCACACTGACGT	CCCCTCCCCGTATCTCCCTTCC
		
*Exon 21*		
First PCR	AGCTTCTTCCCATGATGATCTGTCC	GGCAGCCTGGTCCCTGGTGTC
Second PCR	TCCCATGATGATCTGTCCCTCACA	CAGGAAAATGCTGGCTGACCTAAAG

EGFR=epidermal growth factor receptor; PCR=polymerase chain reaction.

**Table 2:** Patient and tumour characteristics of NSCLCS analysed for *EGFR* mutation and gene copy number

**Characteristics**	**Patients (%)**
Total	84
	
*UICC stage*	
I–IIIA	20 (23.8)
IIIB–IV	58 (69.0)
Recurrence	5 (6.0)
No information	1 (1.2)
	
*Cancer type*	
NSCLC, NOS	26 (31.0)
AC	51 (60.7)
SCC	6 (7.1)
LCNEC	1 (1.2)

AC=adenocarcinoma; EGFR=epidermal growth factor receptor; LCNEC=large cell neuroendocrine carcinoma; NSCLC, NOS=non-small-cell lung carcinoma, not otherwise specified; SCC=squamous cell carcinoma; UICC=International Union Against Cancer.

**Table 3:** Association between *EGFR* mutation and FISH status and clinicopathological characteristics of NSCLCs

	**EGFR mutation^a^**		**EGFR FISH status**	
**Characteristics**	**Present (%)**	**Absent (%)**	**Positive (%)**	**Negative (%)**
Total	6 (7.5)	74 (92.5)	45 (67.2)	22 (32.8)
				
*Gender*				
Male (*n*=55)	2 (3.6)	53 (96.4)	32 (68.1)	15 (31.9)
Female (*n*=25)	4 (16)	21 (84)	13 (65)	7 (35)
*P*	0.07		1.00	
		
*UICC stage*				
I–IIIA	2 (11.1)	16 (88.9)	7 (50)	7 (50)
IIIB–IV	3 (5.4)	53 (94.6)	35 (74.5)	12 (25.5)
Recurrence	0	5	2 (40)	3 (60)
No information	1		1	
*P* (I–IIIA *vs* IIIB–IV)	0.59		0.11	
		
*Cancer type*				
NSCLC, NOS	3 (12.5)	21 (87.5)	12 (66.7)	6 (33.3)
AC	3 (6.1)	46 (93.9)	30 (66.7)	15 (33.3)
SCC	0	6	3 (75)	1 (25)
LCNEC	0	1	0	0

AC=adenocarcinoma; EGFR=epidermal growth factor receptor; FISH=fluorescence *in situ* hybridisation; LCNEC=large cell neuroendocrine carcinoma; NSCLC, NOS=non-small-cell lung carcinoma, not otherwise specified; SCC=squamous cell carcinoma; UICC=International Union Against Cancer.

Criteria for a positive FISH result: presence of high polysomy (⩾4 *EGFR gene* copies per nucleus in ⩾40% of the analysed cancer cells) or of amplification (presence of tight *EGFR gene* clusters and a ratio of *EGFR gene* to chromosome of ⩾2, or ⩾15 EGFR gene copies per nucleus in ⩾10% of the analysed cancer cells).

a The results include two cases in which the *EGFR* mutation was detected on the biopsy specimens.

**Table 4:** NSCLCs with *EGFR* gene mutation: summary of *EGFR* mutation types, FISH results and patient characteristics

**Patient**	**EGFR mutation**	**EGFR FISH result**	**Sex**	**Age (years)**	**UICC stage**	**Diagnosis**	**Smoking history**
1	Exon 19 del E746-A750	Positive amplification	Female	35	IV	NSCLC	No
2	Exon 20 Ins ASV 770–772	Positive amplification	Female	68	IV	AC	Yes
3	Exon 20 F795F	Positive high polysomy	Male	71	—a	AC	—a
4	Exon 21 L858R	Positive high polysomy	Female	49	IV	NSCLC	Yes
5	Exon 21 R832H	Negative	Male	66	IIIA	NSCLC	Yes
6	Exon 21 R837R	Negative	Female	81	IIA	NSCLC	No

AC=adenocarcinoma; del=deletion; EGFR=epidermal growth factor receptor; FISH=fluorescence *in situ* hybridisation; ins=insertion; NSCLC=non-small-cell lung carcinoma; UICC=International Union Against Cancer.

a No information.

**Table 5:** Association between *EGFR* FISH results in the cytological specimens and matched biopsy specimens from all NSCLCs (*n*=33) and from the subgroup of primary NSCLCs without specimens from regional lymph node metastases (*n*=26)

	**Cytological specimens (%)**	**Biopsy specimens (%)**	***P*-value**
*Total*	33	33	
FISH positive	21 (63.6)	8 (24.2)	
High polysomy	19 (57.6)	5 (15.1)	
Amplification	2 (6)	3 (9.1)	
FISH negative	12 (36.4)	25 (75.8)	
			<0.01^a^
*Primary tumour* b	26	26	
FISH positive	16 (61.5)	7 (26.9)	
High polysomy	14 (53.8)	4 (15.4)	
Amplification	2 (7.7)	3 (11.5)	
FISH negative	10 (38.5)	19 (73.1)	
			0.01^a^

EGFR=epidermal growth factor recepetor gene; FISH=fluorescence *in situ* hybridisation; NSCLC=non-small-cell lung carcinoma.

a *P*-value for difference in FISH positivity between cytology and biopsy.

b Both cytology and biopsy from the primary tumour site.

**Table 6:** Comparison of published EGFR FISH results using the criteria proposed by Cappuzzo *et al* (2005) with present data

**Study**	**Number of evaluated NSCLC**	**Number of scored cells**	**FISH positive (%)**	**Balanced (‘high’) polysomy (%)**	**Amplification (%)**
Cappuzzo *et al* (2005) a	102	—b	33	20	13
Tsao *et al* (2005) a	125	33–100	45	34	11
Hirsch *et al* (2005) a	81	⩾100	32	—b	—b
Hirsch *et al* (2006) a	370	—b	31	17	14
Jeon *et al* (2006) a	262	—b	30.2	19.1	11.1
Ichihara *et al* (2007) a	75	⩾100	41.3	25.3	16
					
*Present study*					
Histology	33	43–100 (mean: 88.6±17.6)	24.2	15.1	9.1
Cytology	67	12–100 (mean: 66.1±31.6)	67.1	52.2	14.9

EGFR=*epidermal growth factor receptor gene*; FISH=fluorescence *in situ* hybridisation; NSCLC=non-small-cell lung carcinoma.

Criteria for a positive FISH result: presence of high polysomy (⩾4 *EGFR gene* copies per nucleus in ⩾40% of the analysed cancer cells) or of amplification (presence of tight *EGFR gene* clusters and a ratio of *EGFR gene* to chromosome of ⩾2, or ⩾15 *EGFR gene* copies per nucleus in ⩾10% of the analysed cancer cells).

a All cited FISH analyses were performed on histological specimens.

b No information.
